# Supplementary material for: C1 CAGE detects transcription start sites and enhancer activity at single-cell resolution
Source: Nat Commun. 2019 Jan 21;10:360. doi: 10.1038/s41467-018-08126-5 (PMC6341120; doi:10.1038/s41467-018-08126-5)
Supplement: Supplementary file 1 — Supplementary Information [file 41467_2018_8126_MOESM1_ESM.doc]

**C1 CAGE detects transcription start sites and enhancer activity at single-cell resolution**

**Kouno et al.**

**Supplementary Information**

**Supplementary Figures**

Supplementary Figure 1: C1 CAGE and C1 STRT comparison

(a) Percentage of reads which are classified as strand invasion calculated using the findStrandInvaders function of CAGEr using a linker of ‘GGG’. (b, c) distribution of expected and observed spike-in molecules using stat_smooth with method=’gam’ from the ggplot2 library for (b) C1 CAGE and (c) C1 STRT. Source data are provided as a Source Data file.


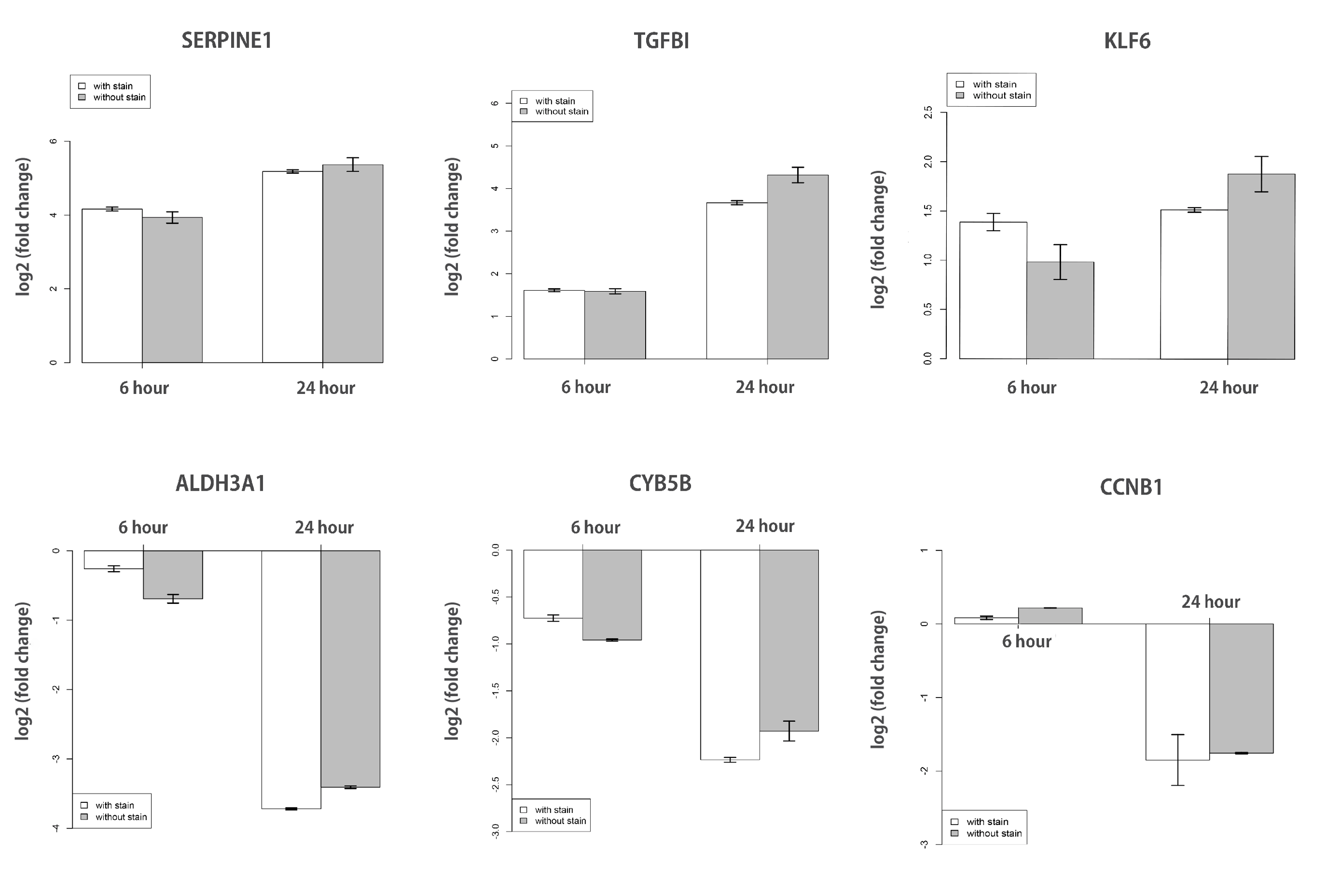


Supplementary Figure 2: Calcein AM staining effect

Fold change for six TGFbeta response genes at 6 and 24 hours compared to 0 hours, with and without Calcein AM staining. Only ALDH3A1 shows significant differences, but in different directions at 6hr and 24hr. Error bars: standard deviations, n=2. Source data are provided as a Source Data file.

Supplementary Figure 3: Species mixing experiment

Fraction of uniquely aligning reads which map to the human genome, in a combined human and mouse genome index, separated by their predicted species based on fluorescence labeling. Source data are provided as a Source Data file.

Supplementary Figure 4: Batch correction

Cells projected onto principal components 1 and 2. Percentage of the variance explained shown in brackets. Cells colored by time point and by replicate. (a) Without batch correction. (b) With batch correction using the replicate and the Calcein stain as the covariates using the limma package removeBatchEffect function. Source data are provided as a Source Data file.

Supplementary Figure 5: Annotation breakdown

18,687 CAGE clusters pass filtering and are included downstream analysis. 2254 of these CAGE clusters could not be annotated, 826 are assigned to FANTOM5 enhancer loci. The remaining clusters are assigned to 9,809 GENCODE genes with the gene categories shown in this figure.

Supplementary Figure 6: C1 CAGE and Bulk CAGE comparison

a,b) Distribution of reads along protein coding genes in a) C1 CAGE, b) bulk CAGE. Protein coding gene length bins are calculated using the union of GENCODE v19 exons assigned to each protein coding gene. c) Features detected in bulk CAGE and C1 CAGE libraries counting reads within FANTOM5 promotor regions. d) Overlap of protein coding genes, enhancer loci and lncRNA genes detected by bulk CAGE and C1 CAGE. Source data are provided as a Source Data file.

Supplementary Figure 7: TSCAN state G2M comparison

Comparisons for the TSCAN states shown in Figure 2d, e. Cells clustered by PCA performed on highly variable genes. Cell cycle phase scores calculated with the cyclone package, cells with high G2M scores are placed in TSCAN state 1. Source data are provided as a Source Data file.

Supplementary Figure 8: Enriched TFs amongst all WGCNA modules

Transcription factor profile contrasting all WGCNA module members against a random GC matched background showing results by (a) Fisher and (b) Z score. Source data are provided as a Source Data file.


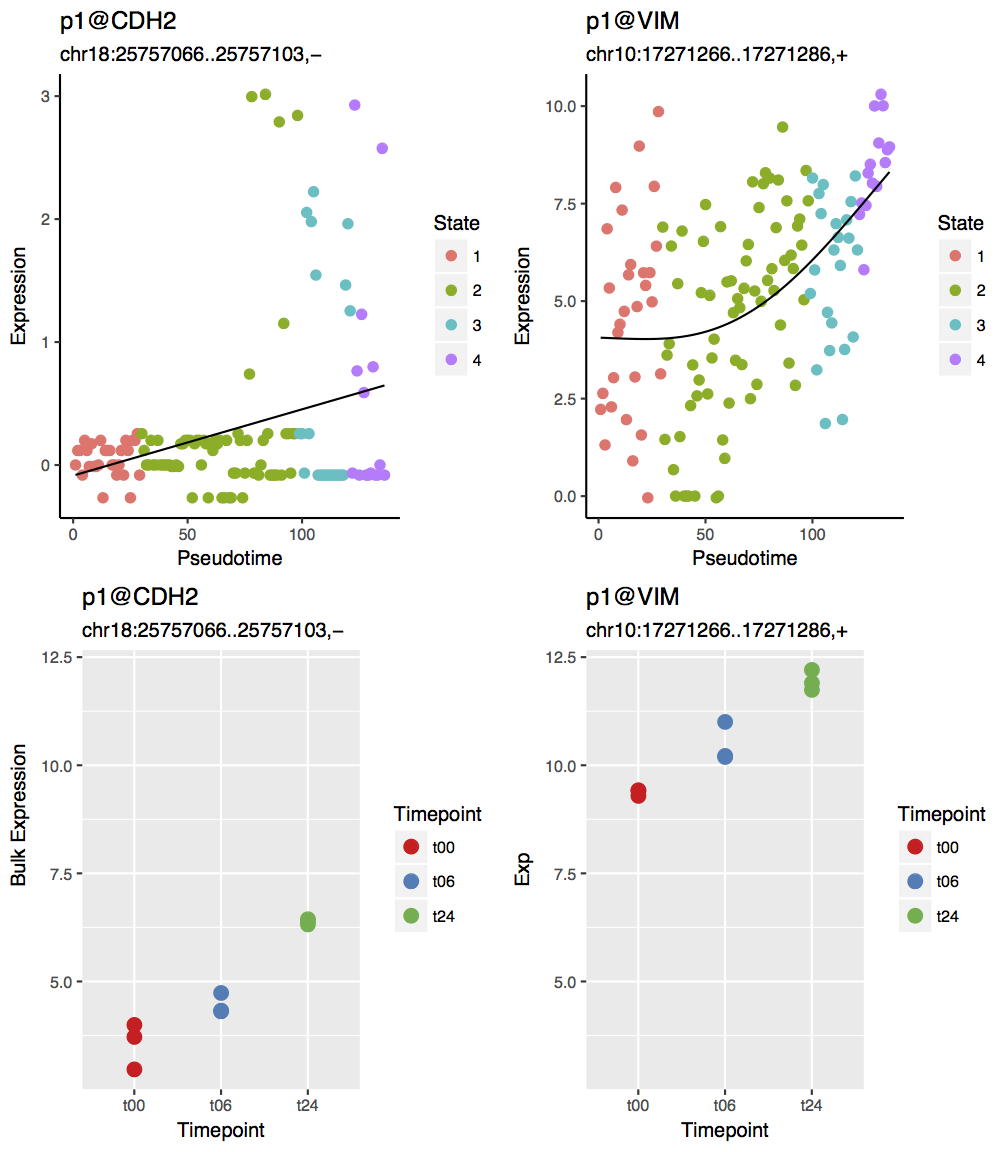


Supplementary Figure 9: Expression profiles for mesenchymal markers CDH2 and VIM.

(upper) Single cell C1 CAGE expression profiles, ordered by pseudotime. (lower) Bulk CAGE expression profiles, grouped by time. Source data are provided in code repository.


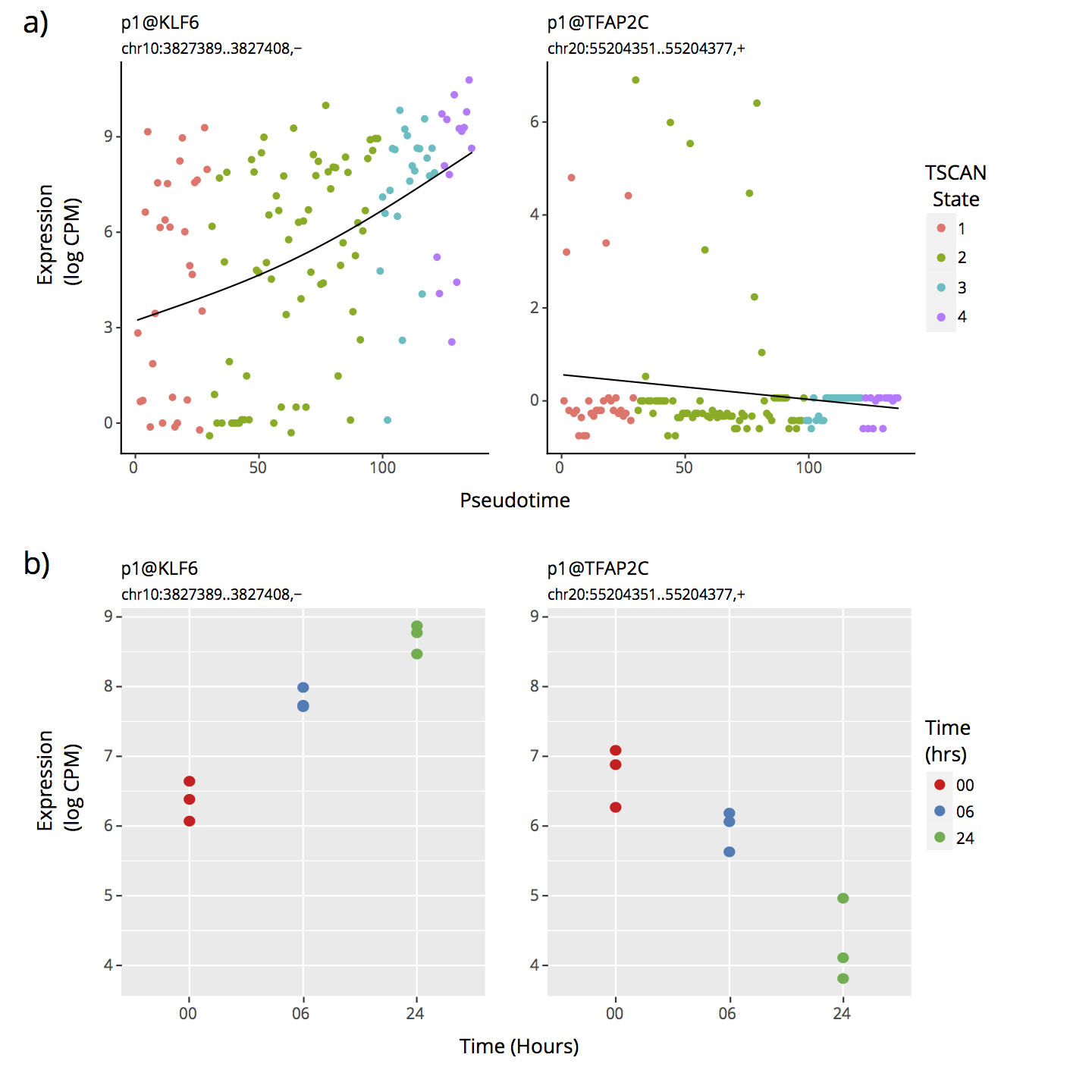


Supplementary Figure 10: Expression profiles for KLF6 and TFAP2C.

(a) Single cell C1 CAGE expression profiles, ordered by pseudotime. (b) Bulk CAGE expression profiles, grouped by time. Source data are provided in code repository.

Supplementary Figure 11: Enhancer thresholds

The number of enhancers with at least n reads in at least x cells for a) all enhancer loci, b) loci with bidirectional reads in pooled cells.

Supplementary Figure 12: Figure4d sorted by pseudotime

Example bidirectional enhancer from main figure 4d, with cells sorted according to pseudotime, demonstrating this is not a time dependent effect. Source data are provided as a Source Data file.


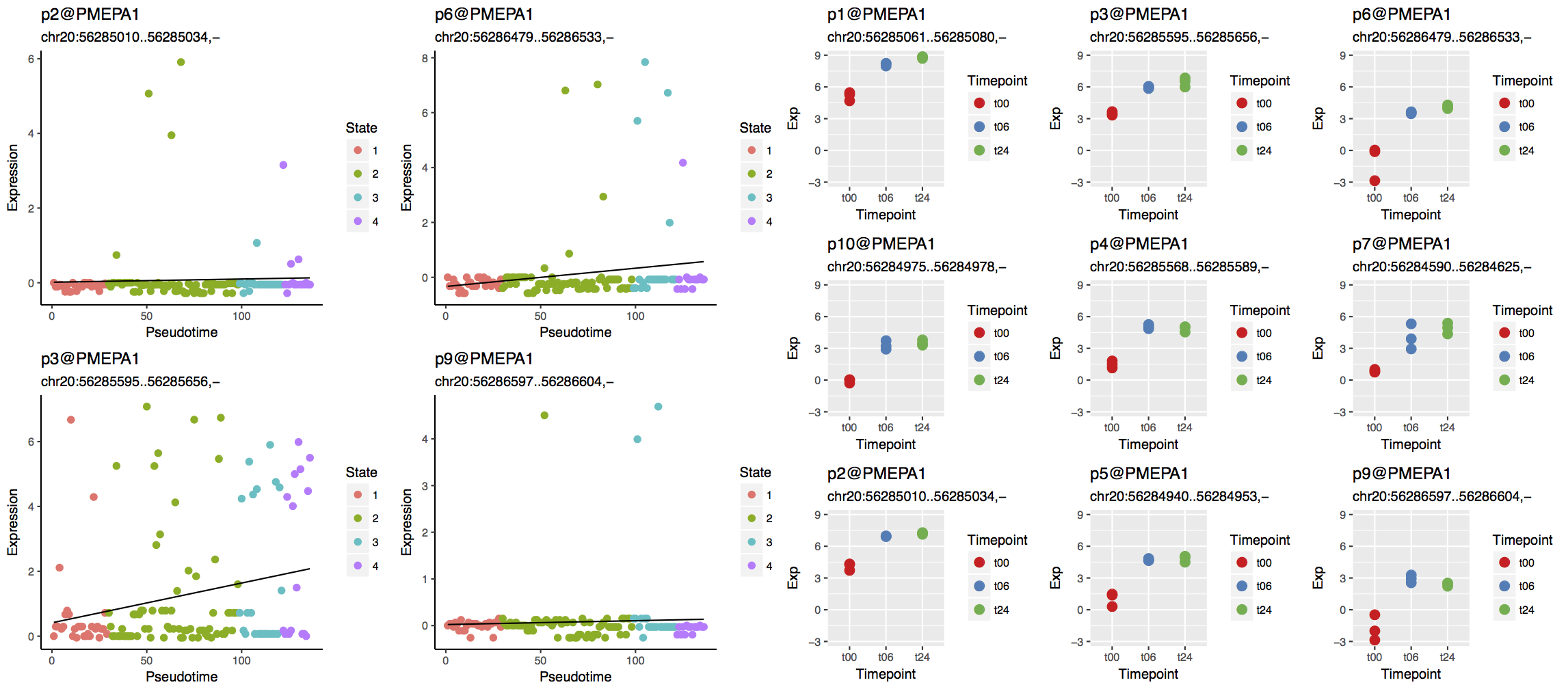


Supplementary Figure 13: Expression profiles for PMEPA1

This gene contains multiple promoters with substantial expression. left) Single cell C1 CAGE expression profiles, ordered by pseudotime. right) Bulk CAGE expression profiles, grouped by time. Source data are provided in code repository.

Supplementary Figure 14: Additional FISH data

Proportion of cells with detected enhancer RNA or intronic gene RNA by (a) FISH, and (b) C1 CAGE. (c) Expression level of genes and enhancer loci in bulk CAGE, Red line: mean. Source data are provided as a Source Data file.


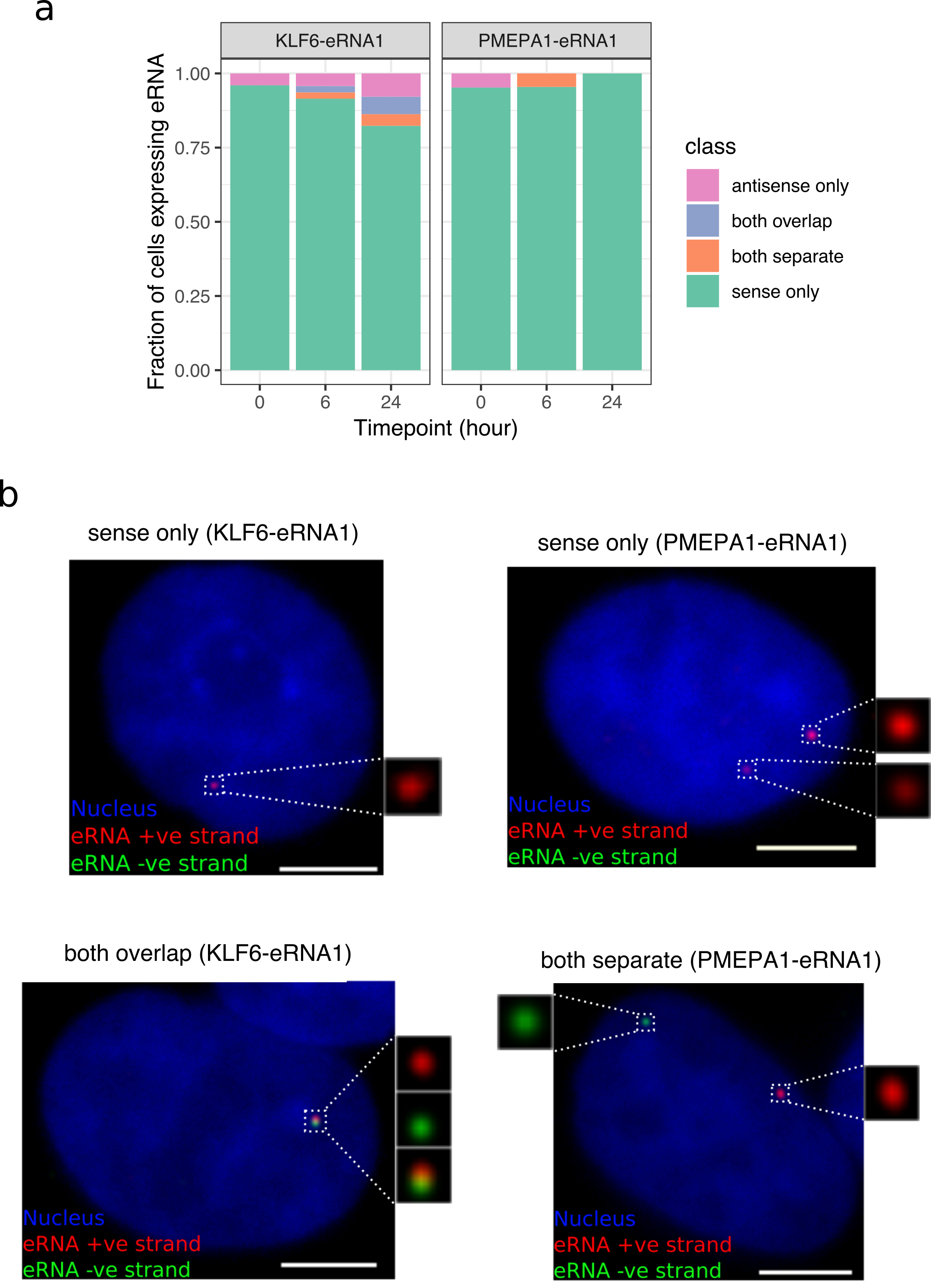


Supplementary Figure 15: FISH directionality and overlap

(a) Strand specificity detected by FISH when each strand is targeted by a different color. (b) Representative FISH images. Bar = 5 μm. n=100 per time point. Source data are provided as a Source Data file.

**Supplementary Tables**

Supplementary Table 1: Script Hub methods

List of the “Gene Expression” protocols available in Fluidigm's Script Hub system as of April 16th, 2018.

Supplementary Table 2: GSEA results

Significant Hallmark gene sets from Camera gene set enrichment analysis testing for differential expression between TSCAN state 3 and 4 based on CAGE clusters from WGCNA Early Responders and Late Responders modules.

| **primer name** | **sequence** |
| --- | --- |
| Reverse transcription primer | TCGTCGGCAGCGTCAGATGTGNNNNNN |
| Template-switching oligonucleotide | TCGTCGGCAGCGTCAGATGTGTATAAGAGACAGNNNNNNNNTATA(rG)(rG)(rG) |
| PCR primer | TCGTCGGCAGCGTCAGATGTG |
| dir#501 | AATGATACGGCGACCACCGAGATCTACACCTCTCTATTCGTCGGCAGCGTCAGATGTGTATAAGAGACAG |
| dir#502 | AATGATACGGCGACCACCGAGATCTACACTATCCTCTTCGTCGGCAGCGTCAGATGTGTATAAGAGACAG |
| dir#503 | AATGATACGGCGACCACCGAGATCTACACGTAAGGAGTCGTCGGCAGCGTCAGATGTGTATAAGAGACAG |
| dir#504 | AATGATACGGCGACCACCGAGATCTACACACTGCATATCGTCGGCAGCGTCAGATGTGTATAAGAGACAG |
| dir#505 | AATGATACGGCGACCACCGAGATCTACACAAGGAGTATCGTCGGCAGCGTCAGATGTGTATAAGAGACAG |
| dir#506 | AATGATACGGCGACCACCGAGATCTACACCTAAGCCTTCGTCGGCAGCGTCAGATGTGTATAAGAGACAG |
| dir#507 | AATGATACGGCGACCACCGAGATCTACACCGTCTAATTCGTCGGCAGCGTCAGATGTGTATAAGAGACAG |
| dir#508 | AATGATACGGCGACCACCGAGATCTACACTCTCTCCGTCGTCGGCAGCGTCAGATGTGTATAAGAGACAG |
| dir#509 | AATGATACGGCGACCACCGAGATCTACACTCGACTAGTCGTCGGCAGCGTCAGATGTGTATAAGAGACAG |
| dir#510 | AATGATACGGCGACCACCGAGATCTACACTTCTAGCTTCGTCGGCAGCGTCAGATGTGTATAAGAGACAG |
| dir#511 | AATGATACGGCGACCACCGAGATCTACACCCTAGAGTTCGTCGGCAGCGTCAGATGTGTATAAGAGACAG |
| dir#512 | AATGATACGGCGACCACCGAGATCTACACGCGTAAGATCGTCGGCAGCGTCAGATGTGTATAAGAGACAG |
| dir#513 | AATGATACGGCGACCACCGAGATCTACACCTATTAAGTCGTCGGCAGCGTCAGATGTGTATAAGAGACAG |
| dir#514 | AATGATACGGCGACCACCGAGATCTACACAAGGCTATTCGTCGGCAGCGTCAGATGTGTATAAGAGACAG |
| dir#515 | AATGATACGGCGACCACCGAGATCTACACGAGCCTTATCGTCGGCAGCGTCAGATGTGTATAAGAGACAG |
| dir#516 | AATGATACGGCGACCACCGAGATCTACACTTATGCGATCGTCGGCAGCGTCAGATGTGTATAAGAGACAG |
| N701 | CAAGCAGAAGACGGCATACGAGATTCGCCTTAGTCTCGTGGGCTCGG |
| N702 | CAAGCAGAAGACGGCATACGAGATCTAGTACGGTCTCGTGGGCTCGG |
| N703 | CAAGCAGAAGACGGCATACGAGATTTCTGCCTGTCTCGTGGGCTCGG |
| N704 | CAAGCAGAAGACGGCATACGAGATGCTCAGGAGTCTCGTGGGCTCGG |
| N705 | CAAGCAGAAGACGGCATACGAGATAGGAGTCCGTCTCGTGGGCTCGG |
| N706 | CAAGCAGAAGACGGCATACGAGATCATGCCTAGTCTCGTGGGCTCGG |
| N707 | CAAGCAGAAGACGGCATACGAGATGTAGAGAGGTCTCGTGGGCTCGG |
| N710 | CAAGCAGAAGACGGCATACGAGATCAGCCTCGGTCTCGTGGGCTCGG |
| N711 | CAAGCAGAAGACGGCATACGAGATTGCCTCTTGTCTCGTGGGCTCGG |
| N712 | CAAGCAGAAGACGGCATACGAGATTCCTCTACGTCTCGTGGGCTCGG |
| N714 | CAAGCAGAAGACGGCATACGAGATTCATGAGCGTCTCGTGGGCTCGG |
| N715 | CAAGCAGAAGACGGCATACGAGATCCTGAGATGTCTCGTGGGCTCGG |

Supplementary Table 3: Primer sequences used in the C1 CAGE method.
