# Supplementary material for: C1 CAGE detects transcription start sites and enhancer activity at single-cell resolution
Source: Nat Commun. 2019 Jan 21;10:360. doi: 10.1038/s41467-018-08126-5 (PMC6341120; doi:10.1038/s41467-018-08126-5)
Supplement: Supplementary file 3 — Description of Additional Supplementary Files [file 41467_2018_8126_MOESM3_ESM.pdf]

Description of Additional Supplementary Files

File Name: Supplementary Data 1
Description: Lists of FISH probes used.

File Name: Supplementary Data 2
Description: TGF-β time-course expression table.
