# Supplementary material for: C1 CAGE detects transcription start sites and enhancer activity at single-cell resolution
Source: Nat Commun. 2019 Jan 21;10:360. doi: 10.1038/s41467-018-08126-5 (PMC6341120; doi:10.1038/s41467-018-08126-5)
Supplement: Supplementary file 7 — Reporting Summary [file 41467_2018_8126_MOESM7_ESM.pdf]

natureresearch

Corresponding author(s): Jay W. Shin, Charles Plessy

Reporting Summary

Nature Research wishes to improve the reproducibility of the work that we publish. This form provides structure for consistency and transparency in reporting. For further information on Nature Research policies, see Authors & Referees and the Editorial Policy Checklist.

Statistical parameters

When statistical analyses are reported, confirm that the following items are present in the relevant location (e.g. figure legend, table legend, main text, or Methods section).

n/a	Confirmed	
☐	☒	The exact sample size (*n*) for each experimental group/condition, given as a discrete number and unit of measurement
☐	☒	An indication of whether measurements were taken from distinct samples or whether the same sample was measured repeatedly
☐	☒	The statistical test(s) used AND whether they are one- or two-sided<br>*Only common tests should be described solely by name; describe more complex techniques in the Methods section.*
☒	☐	A description of all covariates tested
☐	☒	A description of any assumptions or corrections, such as tests of normality and adjustment for multiple comparisons
☐	☒	A full description of the statistics including central tendency (e.g. means) or other basic estimates (e.g. regression coefficient) AND variation (e.g. standard deviation) or associated estimates of uncertainty (e.g. confidence intervals)
☐	☒	For null hypothesis testing, the test statistic (e.g. *F*, *t*, *r*) with confidence intervals, effect sizes, degrees of freedom and *P* value noted<br>*Give P values as exact values whenever suitable.*
☒	☐	For Bayesian analysis, information on the choice of priors and Markov chain Monte Carlo settings
☒	☐	For hierarchical and complex designs, identification of the appropriate level for tests and full reporting of outcomes
☐	☒	Estimates of effect sizes (e.g. Cohen's *d*, Pearson's *r*), indicating how they were calculated
☐	☒	Clearly defined error bars<br>*State explicitly what error bars represent (e.g. SD, SE, CI)*

Our web collection on statistics for biologists may be useful.

Software and code

Policy information about availability of computer code

Data collection	Software and analysis methods used are described in the online methods section and code repository
Data analysis	Software and analysis methods used are described in the online methods section and code repository

For manuscripts utilizing custom algorithms or software that are central to the research but not yet described in published literature, software must be made available to editors/reviewers upon request. We strongly encourage code deposition in a community repository (e.g. GitHub). See the Nature Research guidelines for submitting code & software for further information.

Data

Policy information about availability of data

All manuscripts must include a data availability statement. This statement should provide the following information, where applicable:

- Accession codes, unique identifiers, or web links for publicly available datasets
- A list of figures that have associated raw data
- A description of any restrictions on data availability

C1 CAGE sequence data from this study have been submitted to DDBJ (Project ID: PRJDB5282, Sample ID: SAMD00066188 - SAMD00066475). Alignments were uploaded to the ZENBU genome browser (Severin et al, 2014, PMID 24727769) and a default view is available at http://fantom.gsc.riken.jp/zenbu/gLyphs/#config=NMT9yTLnH59gIVssI9WRfD. In these two submissions the libraries numbered 1, 2 and 3 in this manuscript are numbered 4, 5 and 6, respectively, for

historical reasons

Field-specific reporting

Please select the best fit for your research. If you are not sure, read the appropriate sections before making your selection.

☒ Life sciences ☐ Behavioural & social sciences ☐ Ecological, evolutionary & environmental sciences

For a reference copy of the document with all sections, see nature.com/authors/policies/ReportingSummary-flat.pdf

Life sciences study design

All studies must disclose on these points even when the disclosure is negative.

Sample size	Sample size within each replicate was determined by the technical limitations of the C1 machine. No sample-size calculations were performed.
Data exclusions	Excluded samples were determined as listed in Online methods and labeled in the code repository.
Replication	Three replicates of the time course experiment were performed.
Randomization	Different color combinations of time points were allocated to each replicate to allow for the minimization of batch effects via statistical correction.
Blinding	Investigators were not blinded to group allocations.

Reporting for specific materials, systems and methods

Materials & experimental systems

n/a	Involved in the study	
☒	☐	Unique biological materials
☒	☐	Antibodies
☐	☒	Eukaryotic cell lines
☒	☐	Palaeontology
☒	☐	Animals and other organisms
☒	☐	Human research participants

Methods

n/a	Involved in the study	
☒	☐	ChIP-seq
☒	☐	Flow cytometry
☒	☐	MRI-based neuroimaging

Eukaryotic cell lines

Policy information about cell lines

Cell line source(s)	A549 cells (ATCC CCL 185)
Authentication	Cell lines were not authenticated.
Mycoplasma contamination	Transcriptome alignment of the C1 positive controls against 79 reference genomes of Mycoplasma or Acholeplasma, including Mycoplasma hominis, confirmed the absence of contamination.
Commonly misidentified lines (See ICLAC register)	*Name any commonly misidentified cell lines used in the study and provide a rationale for their use.*
